# A Game-Based Rehabilitation System for Upper-Limb Cerebral Palsy: A Feasibility Study

**DOI:** 10.3390/s20082416

**Published:** 2020-04-24

**Authors:** Mohammad I. Daoud, Abdullah Alhusseini, Mostafa Z. Ali, Rami Alazrai

**Affiliations:** 1 Department of Computer Engineering, German Jordanian University, Amman 11180, Jordan; abdullah.h.alhusseini@gmail.com (A.A.); rami.azrai@gju.edu.jo (R.A.); 2 Department of Computer Information Systems, Jordan University of Science and Technology, Irbid 22110, Jordan; mzali@just.edu.jo

**Keywords:** cerebral palsy, game-based rehabilitation exercises, computerized assessment methods, motion tracking sensors, Kinect sensor

## Abstract

Game-based rehabilitation systems provide an effective tool to engage cerebral palsy patients in physical exercises within an exciting and entertaining environment. A crucial factor to ensure the effectiveness of game-based rehabilitation systems is to assess the correctness of the movements performed by the patient during the game-playing sessions. In this study, we propose a game-based rehabilitation system for upper-limb cerebral palsy that includes three game-based exercises and a computerized assessment method. The game-based exercises aim to engage the participant in shoulder flexion, shoulder horizontal abduction/adduction, and shoulder adduction physical exercises that target the right arm. Human interaction with the game-based rehabilitation system is achieved using a Kinect sensor that tracks the skeleton joints of the participant. The computerized assessment method aims to assess the correctness of the right arm movements during each game-playing session by analyzing the tracking data acquired by the Kinect sensor. To evaluate the performance of the computerized assessment method, two groups of participants volunteered to participate in the game-based exercises. The first group included six cerebral palsy children and the second group included twenty typically developing subjects. For every participant, the computerized assessment method was employed to assess the correctness of the right arm movements in each game-playing session and these computer-based assessments were compared with matching gold standard evaluations provided by an experienced physiotherapist. The results reported in this study suggest the feasibility of employing the computerized assessment method to evaluate the correctness of the right arm movements during the game-playing sessions.

## 1. Introduction

Cerebral palsy refers to a group of non-progressive neurological disorders that begin at early childhood and can lead, among other limitations, to various degrees of motor impairments, physical disability, postural control, and coordination deficits [1,2]. The worldwide rate of cerebral palsy is around 1 per 500 live births, which makes cerebral palsy the most frequent cause of motor disability in childhood [3]. In fact, many children with cerebral palsy suffer from motor impairments in their upper limbs, which lead to significant impact on their independence in activities of daily living, quality of life, social interaction, and functional abilities [4].

Presently, no cure has been discovered for cerebral palsy [5]. Therefore, the treatment of cerebral palsy is often focused on avoiding complications, enhancing functional independence, managing symptoms, improving motor capabilities, and strengthening weak muscles [6]. One of the most effective and common rehabilitation treatment approaches for patients with cerebral palsy is to participate in task-specific, intensive, and repetitive physical exercises to improve the motor capabilities and reduce the potential of further complications [7,8]. Nevertheless, boredom, lack of motivation, the limited available resources, and high costs are among the key barriers that prevent patients with cerebral palsy from participating in rehabilitation physical exercises as recommended [9,10]. To address the aforementioned limitations, many research groups proposed the use of computer games as a complementary tool for conventional cerebral palsy rehabilitation therapy, with particular focus on children patients [11,12,13,14,15,16]. The use of computer games in the rehabilitation process has the potential to motivate the patients and increase the frequency and duration of the exercises [9,17,18].

Studies that investigated the use of computer games for cerebral palsy rehabilitation can be broadly classified into two main groups [13,19]. The first group is focused on using off-the-shelf computer games in cerebral palsy rehabilitation, while the second group proposed the use of bespoke computer games that are specifically designed for cerebral palsy rehabilitation [13,19]. Several studies from the first group employed off-the-shelf games that run on the Nintendo Wii game playing system (Nintendo Co., Kyoto, Japan), such as [11,20,21], the Microsoft Kinect system (Microsoft Corporation, Redmond, WA, USA), such as [10,16,22,23], and the Sony PlayStation system (Sony Interactive Entertainment, San Mateo, CA, USA), such as [24]. The use of off-the-shelf games has the advantage of offering high-quality gaming experience for cerebral palsy children at low cost. Nevertheless, such games are designed for typically developing subjects rather than persons with limited motor abilities [11,21]. Hence, the use of off-the-shelf games for cerebral palsy rehabilitation might be limited due to their high complexity and difficulty, the restricted customization options that prevent their adaptation for the rehabilitation process, and the lack of specialized assessment tools to evaluate the performance of the cerebral palsy children during the game-playing sessions [13,19].

Bespoke computer games that are specifically developed for cerebral palsy rehabilitation provide several advantages, including the ability to target physical rehabilitation exercises and the capability of customizing the games to meet the needs of the patients. In fact, one of the widely used commercial bespoke game-based rehabilitation systems is the Immersive Rehabilitation Exercise system (IREX system, GestureTek Health Inc., Toronto, ON, Canada) [25]. The IREX system employs a camera to capture real-time pictures of the patient and embeds the pictures into virtual reality gaming environment. The patient interacts with the virtual reality environment using motion tracking sensors. The study by Bryanton et al. [17] evaluated the use of the IREX system for cerebral palsy rehabilitation, and the results indicated the potential of employing the system to engage children with cerebral palsy in physical exercises within an exciting environment. Despite the improved capability of the IREX system, the high costs associated with the system might restrict its deployment at home as well as therapy centers that have limited budget [26].

Several previous studies investigated the development of non-commercial, low-cost, and effective bespoke computer games to support the rehabilitation of cerebral palsy children. Detailed review about these previous studies is presented in [25]. In fact, a large group of these non-commercial bespoke rehabilitation games employed the Microsoft Kinect sensor for tracking the skeleton joints of the participant to enable him/her to interact with the games [25]. For example, the study in [27] presented a set of Kinect-based games that are developed to engage patients with motor disabilities, including cerebral palsy patients, in physical exercises. Furthermore, several recent studies, such as [13,19,28], proposed Kinect-based games for training the limbs, particularly the upper limbs, of cerebral palsy children. The results reported in these studies suggested the feasibility of using non-commercial, low-cost, bespoke rehabilitation games as adjunct to conventional rehabilitation programs to actively engage cerebral palsy children in physical exercises. Nevertheless, a crucial requirement to enable the use of bespoke computer games as an effective complementary tool for cerebral palsy rehabilitation is to assess the correctness of the movements performed by the patients during the game-playing sessions [13].

The current study contributes to the ongoing efforts to improve the rehabilitation of cerebral palsy by proposing a non-commercial, low-cost, bespoke rehabilitation system. The proposed system aims to engage patients with upper-limb cerebral palsy in game-based physical exercises that target the right arm and assess the correctness of the right arm movements performed during the playing sessions. In particular, the proposed system includes three game-based rehabilitation exercises that are designed under the supervision of a specialized physiotherapist. Human interaction with the game-based exercises is enabled using the Microsoft Kinect sensor (particularly the Kinect v2 sensor). The correctness of the right arm movements during each game-playing session is evaluated using a custom-made computerized assessment method that analyzes the tracking data acquired by the Kinect sensor. To evaluate the performance of the proposed computerized assessment method, two groups of participants volunteered to participate in the three game-based rehabilitation exercises, where the first group included six cerebral palsy children and the second group included twenty typically developing subjects. For every participant, the computerized assessment method was employed to assess the correctness of the right arm movements in each game-playing session and the obtained computer-based assessment was compared with matching gold standard evaluation that is provided by an experienced physiotherapist. The results reported in the current study demonstrate the capability of the proposed assessment method to achieve effective evaluation of the right arm movements during the game-playing sessions.

The remainder of the paper is organized as follows. Section 2 provides an overview of the proposed rehabilitation system as well as detailed description of the three game-based rehabilitation exercises and the computerized assessment method. Section 3 describes the experimental evaluations and results, including the two groups of participants, the experimental protocol, the performance evaluation metrics, and the results obtained for both the cerebral palsy children and the typically developing subjects. The discussion and conclusion are provided in Section 4.

## 2. Materials and Methods

### 2.1. Overview of the Proposed Rehabilitation System

**Figure 1:**
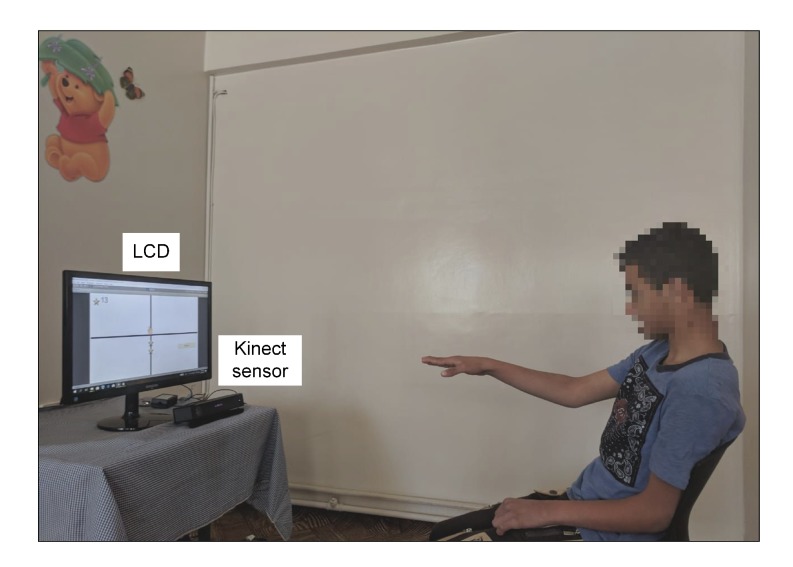
A cerebral palsy patient playing one of the game-based rehabilitation exercises provided by our proposed system.

The hardware component of our proposed bespoke game-based rehabilitation system consists of a personal computer connected to a 19” liquid crystal display (LCD) and a Microsoft Kinect sensor. The personal computer is employed to perform the computational tasks, the LCD is used to display the game-based rehabilitation exercises, and Kinect sensor is employed to track the skeleton joints of the participant during each game-playing session. The participant was asked to perform the game-based rehabilitation exercises while seating on a chair or a wheelchair. Moreover, the LCD and the Kinect sensor were placed on a table with adjustable height to enable the participant to perform the game-based rehabilitation exercises in a comfortable manner. Figure 1 shows a cerebral palsy patient playing one of the game-based rehabilitation exercises provided by the proposed system.

The Kinect sensor used in the current study is the Kinect for Windows v2 sensor. In fact, this sensor provides a low-cost and effective alternative for high cost reference motion tracking systems, such as the Qualisys motion capture system (Qualisys Inc., Gothenburg, Sweden) [29]. The Kinect v2 sensor is equipped with an RGB camera that has a resolution of 1920 × 1080 pixels and an infrared camera that has a resolution of 512 × 424 pixels. Moreover, the sensor can track the 3D positions of twenty five body skeleton joints with a frame rate up to 30 Hz, where each joint position is expressed in the 3D Cartesian coordinate system.

The software component of our proposed system consists of a set of three game-based rehabilitation exercises that target the right arm and a computerized assessment method that assists the correctness of the right arm movements during the game-playing sessions. In fact, the game-based rehabilitation exercises are developed using the C# programming language and the Unity game development platform (Unity Technologies ApS, San Francisco, CA, USA). The computerized assessment method was implemented using MATALB (MathWorks Inc., Natick, MA, USA) and run offline after completing the playing sessions of the game-based rehabilitation exercises to evaluate the correctness of the right arm movements.

**Figure 2:**
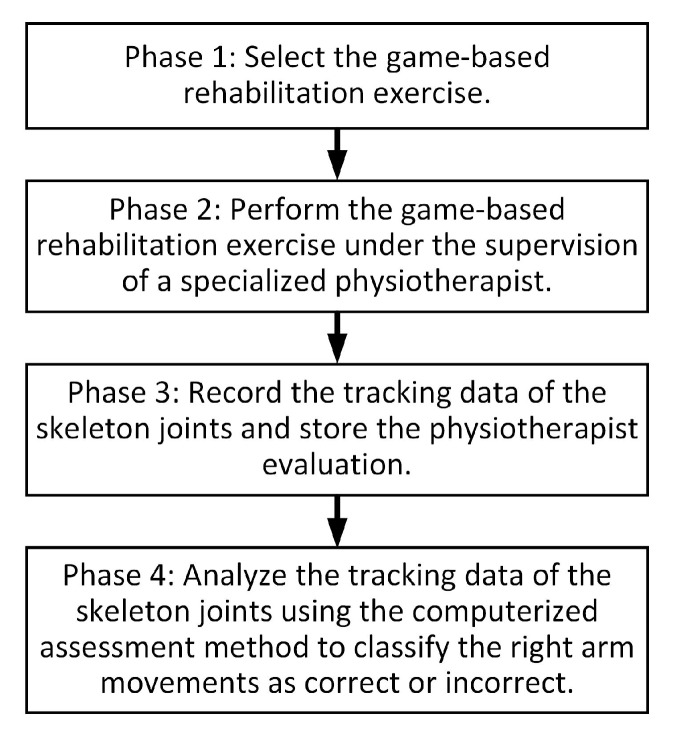
The architecture of the proposed bespoke game-based rehabilitation system.

The architecture of the proposed bespoke game-based rehabilitation system is illustrated in Figure 2. As shown in the figure, the system is composed of four phases. The first phase allows the participant, under the supervision of the specialized physiotherapist, to select the game-based rehabilitation exercise. After selecting the game-based exercise, the system proceeds to the second phase. In the second phase, the participant performs the selected game-based exercise under the supervision and vocal guidance of the physiotherapist. In fact, the vocal guidance of the physiotherapist aims to help the participant to perform the game-based exercise in a correct manner. At the beginning of the game-playing session of the selected game-based exercise, the participant starts the exercise with his/her arms relaxed on his/her side and his/her palms facing towards his/her body. Then, the participant moves his/her right arm to perform the movements needed to play the game-based exercise as described in Section 2.2. It is worth noting that the game-playing session refers to the sequence of right arm movements performed by the participant to play the game-based exercise for a single iteration. During the game-playing session, the skeleton joints of the participant are tracked using the Kinect sensor to enable the participant to interact with the game. Moreover, the physiotherapist is asked to monitor the performance of the participant. The second phase ends when the participant completes the game-playing session of the selected game-based exercise. In the third phase, the tracking data of the participant’s skeleton joints are recorded. Moreover, the physiotherapist is asked to label the right arm movements during the game-playing session as correct or incorrect based on the evaluation criterion described in Section 3.2. In the fourth phase, the recorded tracking data of the participant’s skeleton joints are analyzed using the computerized assessment method, which is described in Section 2.3, to provide a computer-based evaluation of the correctness of the right arm movements during the game-playing session.

### 2.2. The Game-Based Rehabilitation Exercises

The proposed bespoke rehabilitation system includes three game-based exercises that target the right arm. The three game-based exercises are designed under the supervision of a specialized physiotherapist with more than 12 years of experience to increase the range of motion and improve the stability of the right arm. In particular, the three game-based exercises aim to implement the shoulder flexion, shoulder horizontal abduction/adduction, and shoulder adduction physical exercises. Below is a summary of the three game-based exercises.

#### 2.2.1. The Shoulder Flexion Game-Based Exercise

**Figure 3:**
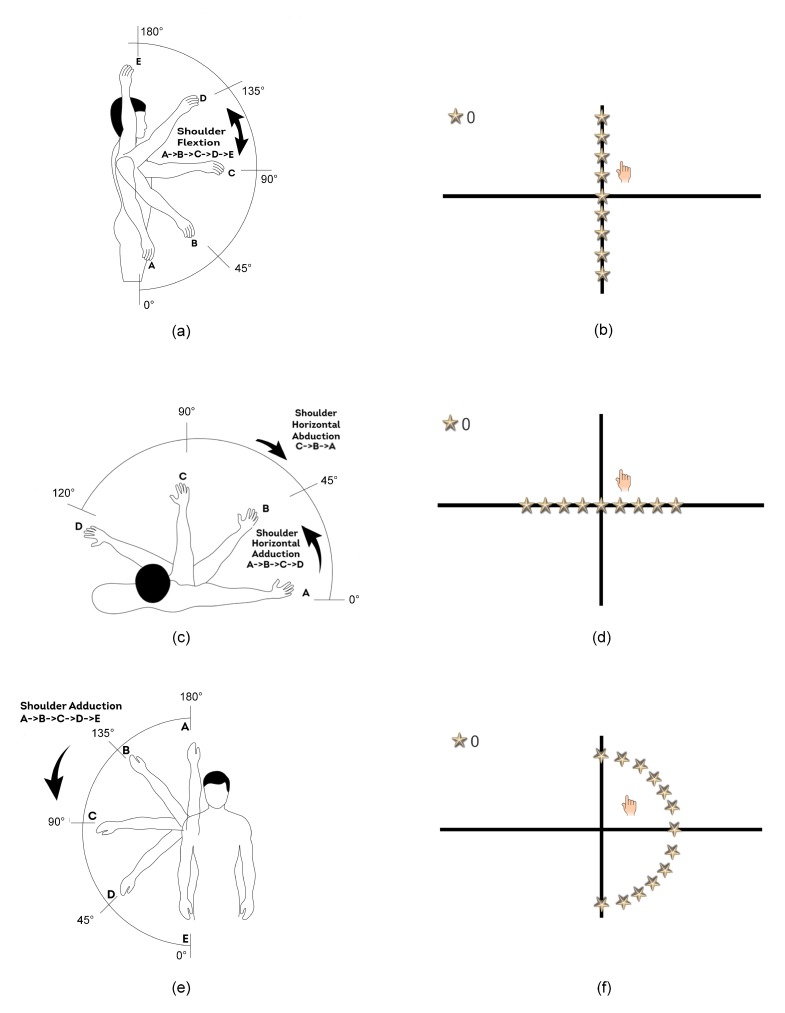
(**a**,**b**): (**a**) The shoulder flexion physical exercise and (**b**) the corresponding shoulder flexion game-based exercise. (**c**,**d**): (**c**) The shoulder horizontal abduction physical exercise followed by the shoulder horizontal adduction physical exercise and (**d**) the corresponding shoulder horizontal abduction/adduction game-based exercise. (**e**,**f**): (**e**) The shoulder adduction physical exercise and (**f**) the corresponding shoulder adduction game-based exercise.

The first game-based exercise aims to engage the participant in the shoulder flexion physical exercise, which is illustrated in Figure 3a. The game-based exercise that is employed to realize the shoulder flexion physical exercise is illustrated in Figure 3b. In this game, a set of nine stars are placed along the central, vertical axis of the screen. The participant is asked to collect the stars using a virtual hand that is controlled using the participant’s right hand through the Kinect sensor. In particular, the participant is asked to lift his/her right arm up from the resting position by his/her side (position A in Figure 3a) to his/her front in which the arm is parallel to the floor (position C in Figure 3a). The participant continues the game by lifting his/her arm up from the front position (position C in Figure 3a) to the straight position above the head (position E in Figure 3a). These movements allow the participant to collect the stars located at the screen. The participant maintains his/her arm in the straight position above the head for a predefined time period, which is between 1 and 2 s. At the end of the game, the participant is asked to move his/her arm down from the straight position above the head (position E in Figure 3a) to the resting position by his/her side (position A in Figure 3a), where this movement allows him/her to collect any remaining stars in the screen. The participant is asked to perform all these movements slowly and in a controllable manner in which the arm is kept straight. Moreover, the trunk should be kept straight during the exercise.

To engage the participant in the game in an enjoyable and fun environment, we have added sound effects to the game, such that a cling sound is played each time a star is collected by the participant. In addition, the score of the game is incremented by one point when the participant collects a star. The sound effect and the process of assigning one point for each collected star is applied for the two game-based exercises described below.

#### 2.2.2. The Shoulder Horizontal Abduction/Adduction Game-Based Exercise

This game-based exercise aims to engage the participant in the shoulder horizontal abduction and shoulder horizontal adduction physical exercises, which are illustrated in Figure 3c. The game-based exercise that is employed to realize these two physical exercises is shown in Figure 3d. In this game, a set of nine stars are placed along the central, horizontal axis of the screen. The participant is asked to collect the stars using a virtual hand that is controlled using the participant’s right hand through the Kinect sensor. At the beginning of the game, the participant is asked to lift his/her arm up from the resting position by his/her side to his/her front in which the arm is parallel to the floor (position C in Figure 3c). The participant maintains his/her arm in the front position for a predefined time period, which is between 1 and 2 s. Then, the participant starts playing the game by moving his/her arm to perform the shoulder horizontal abduction movement (positions C to A in Figure 3c), which allows him/her to collect the stars located at the right half of the screen. After that, the participant is asked to move his/her arm to perform the shoulder horizontal adduction movement (position A to D in Figure 3c), which allows him/her to collect any missing star in the right half of the screen and all stars in the left half of the screen. In fact, the participant is asked to perform all these movements slowly and in a controllable manner in which the arm is kept straight and at the height of the shoulder. Moreover, the trunk should be kept straight during the exercise.

#### 2.2.3. The Shoulder Adduction Game-Based Exercise

This game-based exercise aims to engage the participant in the shoulder adduction physical exercise, which is illustrated in Figure 3e. The game-based exercise that is employed to realize the shoulder adduction physical exercise is illustrated in Figure 3f. In this game, a set of thirteen stars are placed along an arc that is located at the right side of the screen and spans 180°. The participant is asked to collect the stars using a virtual hand that is controlled using the participant’s right arm through the Kinect sensor. At the beginning of the game, the participant is asked to lift his/her arm up from the resting position by his/her side to a straight position above the head (position A in Figure 3e). The participant maintains his/her arm at this position for a predefined time period, which is between 1 and 2 s. Then, the participant starts playing the game by moving his/her arm to perform the shoulder adduction movement (positions A to E in Figure 3e), which allows him/her to collect the stars located in the arc. In fact, the participant is asked to perform this movement slowly and in a controllable manner in which the arm is kept straight. Moreover, the trunk should be kept straight during the exercise.

### 2.3. The Computerized Assessment Method

This subsection describes the computerized assessment method that we have developed to achieve automatic evaluation of the right arm movements during the game-based rehabilitation exercises. In fact, this assessment method is based on spatial-temporal analysis of the 3D tracking data of the participant’s skeleton joints that is acquired by the Kinect sensor during each game-playing session. To develop the computerized assessment method, the Motion-Pose Geometric Descriptor (MPGD), which was originally introduced by Alazrai et al. to model human movements during human-to-human interactions [30] as well as to detect and predict the fall of elderly people [31,32], is extended in the current study to obtain a view-invariant representation of the pose and motion of the right arm. The extended MPGD, denoted by E-MPGD, aims to extract a set of time-varying features that quantify the pose and motion of the participant’s right arm during each game-playing session of the game-based rehabilitation exercises. The extracted features are processed using feature selection and classification analysis to obtain computer-based assessment of the correctness of the right arm movements. In the following subsections, we provide detailed descriptions of the E-MPGD as well as the feature selection and classification analyses employed in the current study.

#### 2.3.1. The Extended Motion-Pose Geometric Descriptor (E-MPGD)

The original formulation of the MPGD [30] used a body-attached 3D coordinate system to compute a set of time-varying features that quantify the pose and motion of the body parts. The body-attached coordinate system is a reference 3D coordinate system that is centered at one of the body joints, denoted by the *origin*, and includes three orthogonal anatomical planes. These anatomical planes are based on the anatomical planes concept described in [33]. A major advantage of the body-attached coordinate system is the capability to obtain view-invariant representation of the 3D positions and movements of the tracked skeleton joints, independently of the location and orientation of the Kinect sensor. Therefore, the locations of the skeleton joints that are tracked by the Kinect sensor are transformed to the body-attached coordinate system to achieve view-invariant analysis of the body movements. The transformed tracking data is then processed to extract a set of time-varying features that describe the pose and motion of the human body.

In the current study, all subjects who participated in the game-based rehabilitation exercises were asked to use their right hands to perform the exercises. Hence, the extended MPGD, denoted by E-MPGD, that we have developed in the current study aims to extend the body-attached coordinate system and the time-varying features of the MPGD to achieve effective representation of the pose and movement of the right arm during the game-based rehabilitation exercises. In the following, we provide detailed description of the body-attached coordinate system and the time-varying features.

##### E-MPGD: The Body-Attached Coordinate System

**Figure 4:**
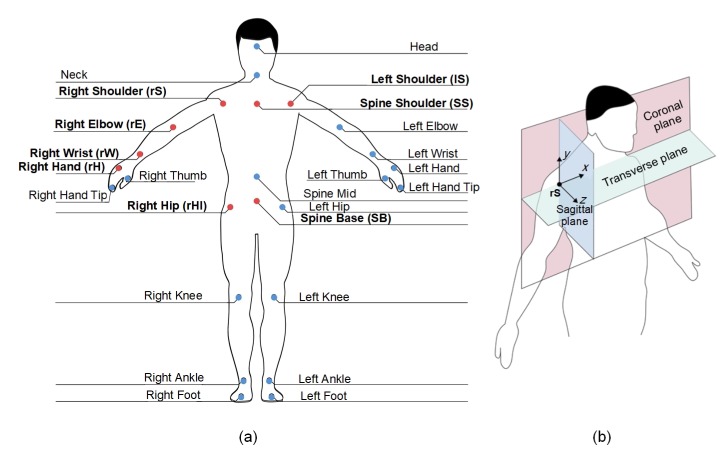
(**a**) The twenty five skeleton joints that are tracked by the Kinect for Windows v2 sensor. (**b**) The body-attached coordinate system of the Extended Motion-Pose Geometric Descriptor (E-MPGD).

The Kinect for Windows v2 sensor employed in the current study has the ability to track the 3D positions of twenty five skeleton joints, which are shown in Figure 4a as red and blue points. In fact, the eight skeleton joints shown as red points, which include the left shoulder (lS) joint, the right shoulder (rS) joint, the spine shoulder (SS) joint, the right hip (rHI) joint, the spine base (SB) joint, the right elbow (rE) joint, the right wrist (rW) joint, and the right hand (rH) joint, are used by the E-MPGD to model the pose and motion of the right arm. The remaining seventeen skeleton joints, which are shown in Figure 4a as blue points, are not considered in the current study as they are not required to model the pose and motion of the right arm.

The body-attached coordinate system of the E-MPGD is configured such that its origin is located at the rS skeleton joint, as shown in Figure 4b. Hence, the 3D locations of the eight skeleton joints considered in the current study, which are shown in Figure 4a as red points, are expressed with respect to the rS joint. In addition, three orthogonal anatomical planes, namely the coronal plane, transverse plane, and sagittal plane, are defined such that these three planes intercept at the origin, as shown in Figure 4b. Each one of these three planes is defined using three non-collinear joints, i.e., joints that are not located along the same line, as described below:

- **The coronal plane (CP):** This plane is defined using the rS, lS, and SB skeleton joints.
- **The transverse plane (TP):** This plane is defined using the rS and lS skeleton joints as well as the $\hat{SS}$ virtual joint. The $\hat{\mathrm{SS}}$ virtual joint is obtained by shifting the SS skeleton joint along the direction orthogonal to the coronal plane, denoted by the positive *z* direction, by a distance of 0.2 m to ensure that the *z* coordinate of the $\hat{SS}$ joint is different than the rS and lS joints.
- **The sagittal plane (SP):** This plane is defined using the rS skeleton joint as well as the $\hat{rS}$ and $\hat{\hat{rS}}$ virtual joints. The $\hat{\mathrm{rS}}$ virtual joint is obtained by shifting the rS skeleton joint along the positive *z* direction by a distance of 0.2 m. The $\hat{\hat{rS}}$ virtual joints is obtained by shifting the rS skeleton joint along the direction orthogonal to the transverse plane, denoted by the negative *y* direction, to match the *y* coordinate of the rHI joint.

##### E-MPGD: The Time-Varying Features

For all game-based rehabilitation exercises, the tracking data acquired by the Kinect v2 sensor during a particular game-playing session is composed of a series of acquisition frames, where each frame includes the *x*, *y*, and *z* coordinates of the twenty five tracked skeleton joints. For every acquisition frame, which is assumed to have a temporal index *k*, the coordinates of the skeleton joints are analyzed to synthesize the body-attached coordinate system, as described before. Moreover, the 3D coordinates of the skeleton joints are transformed to the body-attached coordinate system. After that, the coordinates of the rS (i.e., origin), rE, rW, and rH skeleton joins at frame *k* are analyzed to extract eleven angle-based features and twenty seven joint-based features to quantify the pose and motion of the right arm at that frame. The extraction of the angle-based features is performed by computing three vectors that correspond to the main parts of the right arm. These vectors are the vector that extends from the rS to the rE skeleton joints ($\vec{\mathrm{SE}}$), the vector that extends from the rE to the rW skeleton joints ($\vec{\mathrm{EW}}$), and the vector that extends from the rW to the rH skeleton joints ($\vec{\mathrm{WH}}$). The first nine angle-based features are extracted by computing the angles between each one of these three vectors and the three anatomical planes, i.e., the CP, TP, and SP, at frame *k*. These nine features are denoted by $\theta_{CP}^{\vec{SE}}\left(k\right)$, $\theta_{TP}^{\vec{SE}}\left(k\right)$, $\theta_{SP}^{\vec{SE}}\left(k\right)$, $\theta_{CP}^{\vec{EW}}\left(k\right)$, $\theta_{TP}^{\vec{EW}}\left(k\right)$, $\theta_{SP}^{\vec{EW}}\left(k\right)$, $\theta_{CP}^{\vec{WH}}\left(k\right)$, $\theta_{TP}^{\vec{WH}}\left(k\right)$, and $\theta_{SP}^{\vec{WH}}\left(k\right)$. The descriptions and mathematical formulations of these nine features are provided in Table 1. The remaining two angle-based features are the angle between the vectors $\vec{\mathrm{SE}}$ and $\vec{\mathrm{EW}}$, denoted by $\theta_{\vec{SE}}^{\vec{EW}}\left(k\right)$, and the angle between the vectors $\vec{\mathrm{EW}}$ and $\vec{\mathrm{WH}}$, denoted by $\theta_{\vec{EW}}^{\vec{WH}}\left(k\right)$, at frame *k*. Table 1 provides the descriptions and mathematical formulations of these two features.

The twenty seven joint-based features aim to quantify the motion of the rE, rW, and rH skeleton joints at frame *k*. In particular, nine of these features, which are denoted by $p_{x}^{\mathrm{rE}}\left(k\right)$, $p_{y}^{\mathrm{rE}}\left(k\right)$, $p_{z}^{\mathrm{rE}}\left(k\right)$, $p_{x}^{\mathrm{rW}}\left(k\right)$, $p_{y}^{\mathrm{rW}}\left(k\right)$, $p_{z}^{\mathrm{rW}}\left(k\right)$, $p_{x}^{\mathrm{rH}}\left(k\right)$, $p_{y}^{\mathrm{rH}}\left(k\right)$, $p_{z}^{\mathrm{rH}}\left(k\right)$, evaluate the *x*, *y*, and *z* components of the positions of the rE, rW, and rH skeleton joints at frame *k*. Moreover, nine joint-based features, which are denoted by $v_{x}^{\mathrm{rE}}\left(k\right)$, $v_{y}^{\mathrm{rE}}\left(k\right)$, $v_{z}^{\mathrm{rE}}\left(k\right)$, $v_{x}^{\mathrm{rW}}\left(k\right)$, $v_{y}^{\mathrm{rW}}\left(k\right)$, $v_{z}^{\mathrm{rW}}\left(k\right)$, $v_{x}^{\mathrm{rH}}\left(k\right)$, $v_{y}^{\mathrm{rH}}\left(k\right)$, and $v_{z}^{\mathrm{rH}}\left(k\right)$, quantify the *x*, *y*, and *z* components of the velocities of the rE, rW, and rH skeleton joints at frame *k*. The last nine joint-based features, which are denoted by $a_{x}^{\mathrm{rE}}\left(k\right)$, $a_{y}^{\mathrm{rE}}\left(k\right)$, $a_{z}^{\mathrm{rE}}\left(k\right)$, $a_{x}^{\mathrm{rW}}\left(k\right)$, $a_{y}^{\mathrm{rW}}\left(k\right)$, $a_{z}^{\mathrm{rW}}\left(k\right)$, $a_{x}^{\mathrm{rH}}\left(k\right)$, $a_{y}^{\mathrm{rH}}\left(k\right)$, and $a_{z}^{\mathrm{rH}}\left(k\right)$, evaluate the *x*, *y*, and *z* components of the accelerations of the rE, rW, and rH skeleton joints at frame *k*. The descriptions and mathematical formulations of the joint-based features are provided in Table 1.

#### 2.3.2. Features Extraction, Selection, and Classification

Assuming that the tracking data acquired by the Kinect v2 sensor during the game-playing session of a particular participant and a particular game-based exercise is composed of *L* frames and the goal is to classify the right arm movements during this game-playing session as correct or incorrect. Then, for each frame, we extract a feature vector composed of the eleven angle-based features and the twenty seven joint-based features that are summarized in Table 1. This extraction process generates a series of *L* feature vectors, where each vector is composed of the angle- and joint-based features. The series of *L* feature vectors of the game-playing session under consideration is classified to identify if the right arm movements during the game-playing session are performed correctly or incorrectly. The classification process is performed by comparing the series of L feature vectors of the game-playing session under consideration with a set of correct and incorrect gold standard game-playing sessions that are performed by the same participant for the same game-based rehabilitation exercise. Based on this comparison, the game-playing session under consideration can be considered similar to the correct gold standard game-playing sessions, and hence the right arm movements during the game-playing session are classified as correct. Otherwise, the game-playing session under consideration is considered similar to the incorrect gold standard game-playing sessions, and hence the right arm movements during the game-playing session are classified as incorrect.

The comparison process is performed as follows. For the participant and the game-based rehabilitation exercise under consideration, we assume that there is a set of gold standard game-playing sessions, where this set includes $N_{1}$ game-playing sessions that are labeled as *correct* and $N_{2}$ game-playing sessions that are labeled as *incorrect*. The subset of feature vectors series computed for the correct gold standard game-playing sessions is denoted by $GS_{C}=\left\{C\_SFV_{1},C\_SFV_{2},\ldots ,C\_SFV_{N_{1}}\right\}$, where $C\_SFV_{i}$ represents the feature vector of the *i*th correct gold standard game-playing session. Moreover, the subset of feature vectors series computed for the incorrect gold standard game-playing sessions is denoted by $GS_{IC}=\left\{IC\_SFV_{1},IC\_SFV_{2},\ldots ,IC\_SFV_{N_{2}}\right\}$, where $IC\_SFV_{i}$ represents the feature vector of the *i*th incorrect gold standard game-playing session. Assume that the series of feature vectors of the game-playing session under consideration is denoted as SFV. Then, the average distance, $\bar{dist}\left(SFV,GS_{C}\right)$, between SFV and $GS_{C}$ can be calculated as follows:

(1) $$\bar{dist}\left(SFV,GS_{C}\right)=\frac{\sum_{i=1}^{N_{1}}dist\left(SFV,C\_SFV_{i}\right)}{N_{1}},$$

where $dist(SFV,C\_SFV_{i})$ is the distance between SFV and $C\_SFV_{i}$. The computation of $dist(SFV,C\_SFV_{i})$ should consider that fact that the temporal lengths of the game-playing sessions performed by the same participant for a particular game-based rehabilitation exercise varies from time to time due to the intra-personal variations. Hence, the lengths of SFV and $C\_SFV_{i}$ might be different. Moreover, the frames of SFV and $C\_SFV_{i}$ are most likely asynchronous. To address these limitations, the distance between the feature vectors series SFV and the feature vectors series $C\_SFV_{i}$ is computed using the Multidimensional Dynamic Time Warping (MD-DTW) algorithm [34]. In the same manner, the average distance, $\bar{dist}\left(SFV,GS_{IC}\right)$, between SFV and $GS_{IC}$ is computed as follows:

(2) $$\bar{dist}\left(SFV,GS_{IC}\right)=\frac{\sum_{i=1}^{N_{2}}dist\left(SFV,IC\_SFV_{i}\right)}{N_{2}}.$$

where $dist(SFV,IC\_SFV_{i})$ is the distance between SFV and $IC\_SFV_{i}$ and it is calculated using the MD-DTW algorithm. If the value of $\bar{dist}\left(SFV,GS_{C}\right)$ is greater than or equal to $\bar{dist}\left(SFV,GS_{IC}\right)$, then the right arm movements of the game-playing session under consideration are labeled as correct. Otherwise, the right arm movements of the game-playing session under consideration are labeled as incorrect.

The performance of the classification algorithm described above depends on the capability of the eleven angle-based features and the twenty seven joint-based features, i.e., thirty eight features in total, to differentiate between the correct and incorrect right arm movements. However, not all angle- and joint-based features described in Table 1 can be considered effective to classify the right arm movements. Moreover, the importance of these features varies depending the participant and the game-based exercises under consideration. To improve the performance of the classification algorithm, the recursive backward elimination procedure [35] is employed to select the features combination that optimizes the capability of classifying the right arm movements. In the first iteration of the recursive backward elimination procedure, one of the features is eliminated, and the remaining features are used to classify the right arm movements. Then, the feature that is eliminated in the previous step is returned and another feature is eliminated before running the classification. This elimination process is repeated until each individual feature of the thirty eight features is eliminated before running the classification. The feature that its elimination leads to the highest improvement in the classification performance is permanently removed to achieve thirty seven features with improved classification performance. In the second iteration of the recursive backward elimination procedure, each individual feature of the thirty seven features is eliminated and the classification is run using the remaining features. This process is repeated until identifying the feature that its removal leads to the highest improvement in the classification performance. The recursive iterations of the backward elimination procedure continue until reaching an optimized features combination in which the removal of any feature leads to a reduction in the classification performance. In the current study, the recursive backward elimination procedure is applied in a participant-specific, game-specific manner, in which the process of selecting the features has been performed for each participant and each game-based exercise. Hence, the right arm movements associated with a particular participant and a particular game-based exercise could be classified using the combination of optimized features that is computed specifically for the participant and game-based exercise under consideration.

## 3. Experimental Evaluation and Results

### 3.1. Participants

Two groups of participants volunteered to participate in the experiments to evaluate the performance of the proposed computerized assessment method. The first group included children that are diagnosed with cerebral palsy. These children were recruited from the Model School for Cerebral Palsy, Amman, Jordan to participate in the study during the period between October 2018 and January 2019. The following inclusion criteria have been employed: the participant should be diagnosed with cerebral palsy and his/her age should be 10 years or more. Also, the following exclusion criteria have been applied: the participant cannot perform and interact with the three game-based exercises, he/she do not have the potential to achieve the range of motion targeted by the three game-based exercises as illustrated in Figure 3, and his/her cerebral palsy severity level is classified as severe. Based on these inclusion and exclusion criteria, six cerebral palsy children were selected to participate in the study. The mean ± standard deviation age of the children was 12.7±1.2 years. The gender, the cerebral palsy category, and the cerebral palsy severity of these children are summarized in Table 2. The second group of participants included 20 typically developing subjects (10 females and 10 males) who are undergraduate students at the German Jordanian University, Amman, Jordan. The aim of the second group is to generate additional correct and incorrect game-playing sessions for each one of the three game-based rehabilitation exercises. The typically developing subjects volunteered to participate in the study during the period between August 2018 and September 2018. The mean ± standard deviation age of the typically developing subjects is 20.2±1.2 years. The game-based rehabilitation exercises, which are presented in Section 2.2, and the experimental protocol, which is described below, were explained to the participants in both groups. For the cerebral palsy children, which are younger that 18 years, a signed consent form was collected from the parents of the participants. For the typically developing subjects, which are elder than 18 years, a signed consent form was collected from each subject before participating in the experiments. The experimental protocol employed in the current study was approved by the Ethics Committee at the German Jordanian University, Amman, Jordan and the Ethics Committee at the Model School for Cerebral Palsy, Amman, Jordan.

### 3.2. Experimental Protocol

The participants from both groups were asked to perform the three game-based rehabilitation exercises under the supervision of a specialized physiotherapist. For the cerebral palsy children, every participant was asked to play each one of the three game-based rehabilitation exercises and the physiotherapist guided him/her vocally during the game-playing session to do the right arm movements in a correct manner. For both the correct and incorrect game-playing sessions, the participants maintained the trunk straight throughout the game-playing sessions. In each game-playing session, the physiotherapist evaluated the participant’s performance and labeled each session as correct or incorrect based on the following subject-specific evaluation criteria:

• The participant should perform the movements associated with the game-based exercise, as described in Section 2.2 and illustrated in Figure 3, in a correct manner. In particular, compensatory movements should be avoided as much as possible.
• The participant should cover the range of motion targeted by the game-based exercise, which is illustrated in Figure 3, as much as possible.
• The participant should perform the movements associated with the game-based exercise slowly and in a controllable and stable manner in which the right arm is kept straight as much as possible.

For the group of typically developing subjects, every participant was asked to perform the game-based rehabilitation exercises both correctly and incorrectly and at various speeds. In particular, the incorrect movements performed by the typically developing subjects were focused on replicating the motion limitations of the cerebral palsy children, which mainly include the limited range of motion, the unstable movement of the right arm, and performing the right arm movements associated with the game-based rehabilitation exercise in wrong manners. At the end of each game-playing session, the physiotherapist evaluated the performance of the typically developing subjects using the same evaluation criterion employed for the cerebral palsy children to label the game-playing session as correct or incorrect.

For every participant, including the cerebral palsy children and the typically developing subjects, we have collected 30 correct game-playing sessions and 30 incorrect game-playing sessions for each one of the three game-based rehabilitation exercises. Hence, the total number of game-playing sessions that have been collected for each participant is equal to 180. The mean ± standard deviation temporal lengths of the game-playing sessions performed by the six cerebral palsy children during their participation in the shoulder flexion, shoulder horizontal abduction/adduction, and shoulder adduction game-based exercises are equal to 6.8±5.1 s, 7.6±4.4 s, and 11.4±5.0 s, respectively. Moreover, the mean ± standard deviation temporal lengths of the game-playing sessions performed by the twenty typically developing subjects during their participation in the shoulder flexion, shoulder horizontal abduction/adduction, and shoulder adduction game-based exercises are equal to 4.4±1.2 s, 4.9±1.6 s, and 6.2±1.5 s, respectively. For each cerebral palsy child, the process of acquiring the 180 game-playing sessions was carried out over 12 to 16 recording periods, where in each period the participant performed 10 to 15 game-playing sessions. For the typically developing subjects, the process of acquiring the 180 game-playing sessions was carried out over 3 recording periods, where in each recording period the participant performed 60 game-playing sessions. For all participants, the game-playing sessions were separated by relaxation periods with a minimum length of 2 min. Moreover, the recording periods of each participant were performed on different days. The acquisition rate of the Kinect v2 sensor was set to 15 frames per second for all game-playing sessions.

### 3.3. Performance Evaluation

In the current study, we have employed a participant-specific, game-specific approach to evaluate the performance of the proposed computerized assessment method. In this approach, a tenfold cross validation procedure is used to evaluate the capability of the computerized assessment method to classify the right arm movements of every participant during the sixty game-playing sessions that are recorded for each game-based rehabilitation exercise. To carry out the tenfold cross validation procedure, the sixty game-playing sessions are sorted randomly and divided into 10 uniform subsets, such that each subset includes 3 correct and 3 incorrect game-playing sessions. One of these subsets is selected as a testing subset. Moreover, the remaining 9 subsets are combined and employed for constructing the correct and incorrect gold standard game-playing sessions, which are denoted in Section 2.3.2 as $GS_{C}$ and $GS_{IC}$, respectively. The proposed computerized assessment method was used to classify the 3 correct and 3 incorrect game-playing sessions included in the testing subset based on $GS_{C}$ and $GS_{IC}$, as illustrated in Section 2.3.2. Moreover, the process of selecting one of the subsets as a testing subset and the remaining nine subsets as $GS_{C}$ and $GS_{IC}$ subsets has been repeated for nine folds to enable the computerized assessment method to classify each one of the sixty game-playing sessions. After applying the tenfold cross validation procedure, the classifications obtained by the proposed computerized assessment method for the sixty game-playing sessions were compared with the matching gold standard labels using four performance metrics. The first three metrics are the classification accuracy, specificity, and sensitivity [36]. The last metric is Cohen’s kappa coefficient [37,38]. The kappa coefficient evaluates the agreement between the classifications obtained by the proposed computerized assessment method and the gold standard labels provided by the experienced physiotherapist after considering the agreement occurring by chance. The value of kappa coefficient can be interpreted as follows to quantify the agreement level [39]: (0.8−1]= almost perfect agreement, (0.6−0.8]= substantial agreement, (0.4−0.6]= moderate agreement, (0.2−0.4]= fair agreement, (0−0.2]= slight agreement, and <0= poor agreement.

For every participant and each game-based rehabilitation exercise, the evaluation process described above, which includes randomly sorting the sixty game-playing sessions performed by the participant, applying the tenfold cross validation procedure, and evaluating the capability of the proposed computerized assessment method to classify the sixty game-playing sessions, was repeated for ten evaluation repetitions. The mean ± standard deviation values of the accuracy, specificity, sensitivity, and kappa coefficient are computed for the proposed computerized assessment method across the ten evaluation repetitions that are carried out for every participant and each game-based rehabilitation exercise. Furthermore, for every game-based exercise, the mean ± standard deviation values of the four metrics are computed for all subjects in each one of the two groups of participants.

In addition to evaluating the classification performance of the proposed computerized assessment method, we have analyzed the combinations of angle- and joint-based features that are selected using the features selection approach described in Section 2.3.2 to optimize the classification performance. Particularly, for the first group of participants, which includes six cerebral palsy children, we have computed two metrics: the combination-based occurrence frequency of the features (CBOFF) and the participant-based occurrence frequency of the important features (PBOFIF). For a particular game-based exercise, the two metrics are computed by considering the sixty features combinations that are obtained by combining the optimized features combinations of the ten performance evaluation repetitions performed for each one of the six cerebral palsy children. For each feature, the CBOFF is defined as the number of times the feature appears in the sixty optimized features combinations. The PBOFIF aims to evaluate the importance of the features with respect to the six cerebral palsy children. To compute the PBOFIF, we have developed a custom-made criterion to determine if a given feature is important to classify the ten performance evaluation repetitions performed for a particular participant and a particular game-based rehabilitation exercise. In this criterion, if the feature appears more than two times in the optimized features combinations that are obtained for the ten performance evaluation repetitions, then the feature is considered important to classify the game-playing sessions associated with the participant and game-based rehabilitation exercise under consideration. For a particular game-based exercise, the PBOFIF is computed by individually considering each one of the angle- and joint-based features listed in Table 1 and counting the number of participants in which the feature is considered important. The metrics CBOFF and PBOFIF have also been computed for the second group of participants, which includes twenty typically developing subjects. However, the computation was based on the ten performance evaluation repetitions that are carried out for each one of the twenty typically developing subjects. Hence, the total number of optimized features combinations that are obtained for all typically developing subjects is equal to 200.

### 3.4. Results of the Cerebral Palsy Children

The mean ± standard deviation values of the classification accuracy, specificity, and sensitivity as well as kappa coefficient that are obtained using the optimized combinations of angle- and joint-based features for each one of the six cerebral palsy children during their participation in the three game-based exercises are presented in Table 3. As shown in the table, the mean classification accuracy, specificity, and sensitivity values are between 88% and 95% for child 1 and child 2, between 84% and 88% for child 3 and child 4, and between 71% and 80% for child 5 and child 6. The mean kappa coefficient values are between 0.80 and 0.86 for child 1 and child 2, between 0.70 and 0.74 for child 3 and child 4, and between 0.45 and 0.57 for child 5 and child 6. In addition, Table 3 shows that the mean ± standard deviation values of the accuracy, specificity, sensitivity, and kappa coefficient that are obtained for the three game-based exercises by considering all six cerebral palsy children are within the ranges of 83% to 85%, 83% to 86%, 83% to 84%, and 0.66 to 0.70, respectively.

The results reported in Table 3 indicate that the performance of the computerized assessment method can be related to the cerebral palsy category and severity of the six cerebral palsy children, which are provided in Table 2. In particular, the first four children (child 1, child 2, child 3, and child 4) have spastic cerebral palsy that is associated with contracted and stiff muscles [40]. The right arms of these four children do not suffer from shaky or uncoordinated movements. However, child 1, child 2, and child 3 have spastic diplegic cerebral palsy that mainly affects the muscles of the legs, with lower effect on the arms [40]. The disorder severity of child 1 and child 2 is lower than child 3. Moreover, child 4 is diagnosed with moderate spastic hemiplegia cerebral palsy that mainly affects one side of the body [40]. For child 4, the disorder involves both the right arm and right leg, but the right arm is more affected. The last two children, i.e., child 5 and child 6, have ataxia cerebral palsy with moderate severity. The ataxia cerebral palsy disorder is characterized by shaky and uncoordinated movements as well as poor balance that affects all body parts [40]. The shaky and uncoordinated movements of the right arm and the poor balance increase the difficulty of differentiating the correct game-playing sessions from the incorrect ones. Therefore, the classification results reported for child 1 and child 2 are higher than the results obtained for child 3. Moreover, the results obtained for child 3 and child 4 have comparable values. Furthermore, the lowest classification results are reported for child 5 and child 6.

**Figure 5:**
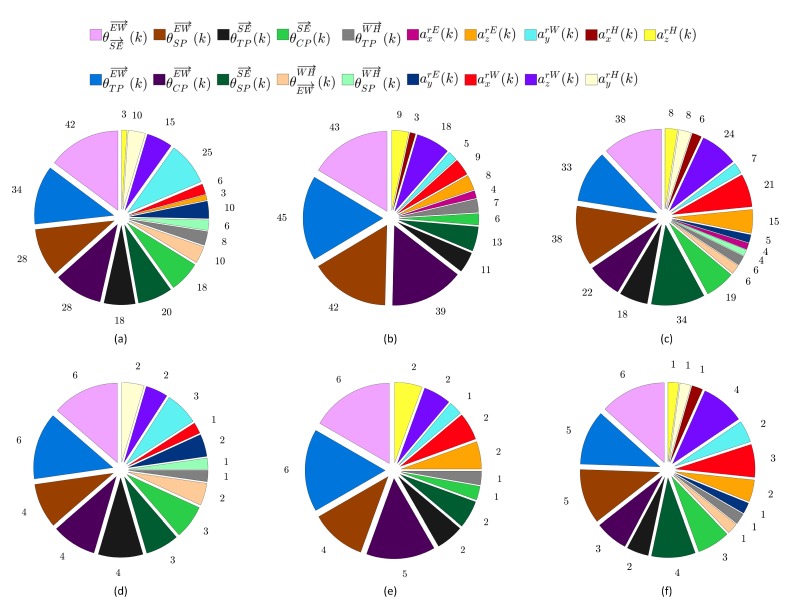
(**a**–**c**) The combination-based occurrence frequency of the features (CBOFF) values computed for the six cerebral palsy children during their participation in (**a**) the shoulder flexion, (**b**) the shoulder horizontal abduction/adduction, and (**c**) the shoulder adduction game-based exercises. (**d**–**f**) The participant-based occurrence frequency of the important features (PBOFIF) values computed for the six cerebral palsy children during their participation in (**d**) the shoulder flexion, (**e**) the shoulder horizontal abduction/adduction, and (**f**) the shoulder adduction game-based exercises.

Figure 5a–c present the CBOFF values computed for the shoulder flexion game-based exercise, the shoulder horizontal abduction/adduction game-based exercise, and the shoulder adduction game-based exercise, respectively. As described in Section 3.3, for a particular game-based exercise, the CBOFF represents the number of times the feature appears in the sixty optimized features combinations that are obtained by combining the optimized features combinations of the ten evaluation repetitions performed for each one of the six cerebral palsy children. Hence, the highest possible CBOFF value is equal to 60. For the three game-based rehabilitation exercises, the CBOFF results indicate that the commonly selected features in the sixty optimized features combinations include mainly the angle-based features that are related to the $\vec{EW}$ and $\vec{SE}$ vectors (i.e., $\theta_{\vec{SE}}^{\vec{EW}}\left(k\right)$, $\theta_{CP}^{\vec{EW}}\left(k\right)$, $\theta_{TP}^{\vec{EW}}\left(k\right)$, $\theta_{SP}^{\vec{EW}}\left(k\right)$, $\theta_{CP}^{\vec{SE}}\left(k\right)$, $\theta_{TP}^{\vec{SE}}\left(k\right)$, and $\theta_{SP}^{\vec{SE}}\left(k\right)$). In addition, the commonly selected features include some position-based features that quantify the acceleration of the skeleton joints. In particular, the acceleration features $a_{y}^{\mathrm{rW}}\left(k\right)$ and $a_{z}^{\mathrm{rW}}\left(k\right)$ are among the commonly selected features for the shoulder flexion game-based exercise, the acceleration feature $a_{z}^{\mathrm{rW}}\left(k\right)$ is among the features that are commonly selected for the shoulder horizontal abduction/adduction game-based exercise, and the acceleration features $a_{z}^{\mathrm{rE}}\left(k\right)$, $a_{x}^{\mathrm{rW}}\left(k\right)$, and $a_{z}^{\mathrm{rW}}\left(k\right)$ are commonly selected for the shoulder adduction game-based exercise. All optimized features combinations obtained for the three game-based rehabilitation exercises do not include any position-based features related to the position or velocity of the skeleton joints.

The PBOFIF values obtained for the shoulder flexion game-based exercise, the shoulder horizontal abduction/adduction game-based exercise, and the shoulder adduction game-based exercise are presented in Figure 5d–f, respectively. In fact, the PBOFIF quantifies the number of cerebral palsy children in which the feature is considered important. Hence, the highest possible PBOFIF value is equal to 6. As described in Section 3.3, for each game-based exercise, the importance of a given feature for a particular cerebral palsy child is determined by analyzing the optimized features combinations of the ten evaluation repetitions performed for the child under considerations. For the three game-based rehabilitation exercises, the important features obtained by considering the PBOFIF metric, which are shown in Figure 5d–f, are close to the commonly selected features obtained by considering the CBOFF metric, which are presented in Figure 5a–c.

### 3.5. Results of the Typically Developing Subjects

For the second group of participants that includes twenty typically developing subjects, the values of the performance metrics computed for the computerized assessment method using the optimized combinations of angle- and joint-based features are presented in Table 4. As shown in the table, the mean classification accuracy, specificity, and sensitivity values that are obtained for the individual typically developing subjects are within the range of 96% to 99%. Moreover, the mean kappa coefficient values achieved for the individual typically developing subjects are between 0.93 and 0.99. When all twenty typically developing subjects are considered, the mean classification accuracy, specificity, and sensitivity values, which are obtained for the three games-based exercises, are within the range of 97% and 98%. Moreover, the mean kappa coefficient values, which are achieved for the three games-based exercises, are between 0.95 and 0.96. These results indicate that the classification performance obtained by the computerized assessment method for the typically developing subjects is higher than the classification performance reported for the cerebral palsy children. This can be attributed to the fact that the typically developing subjects have high control of their right arm movements compared with the cerebral palsy children.

**Figure 6:**
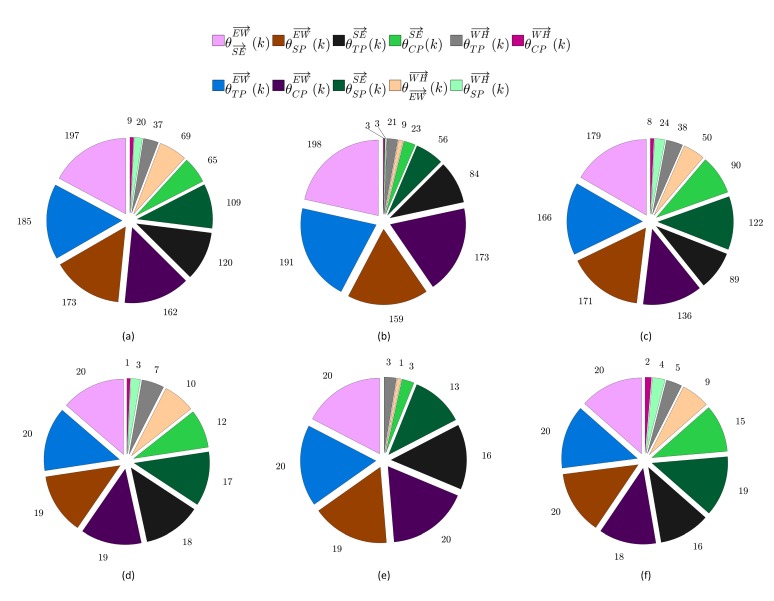
(**a**–**c**) The CBOFF values computed for the twenty typically developing subjects during their participation in (**a**) the shoulder flexion, (**b**) the shoulder horizontal abduction/adduction, and (**c**) the shoulder adduction game-based exercises. (**d**–**f**) The PBOFIF values computed for the twenty typically developing subjects during their participation in (**d**) the shoulder flexion, (**e**) the shoulder horizontal abduction/adduction, and (**f**) the shoulder adduction game-based exercises.

Figure 6a–c show the CBOFF values computed for the twenty typically developing subjects during their participation in the shoulder flexion game-based exercise, the shoulder horizontal abduction/adduction game-based exercise, and the shoulder adduction game-based exercise, respectively. As described in Section 3.3, for a particular game-based exercise, the CBOFF represents the number of times the feature appears in the 200 optimized features combinations that are obtained by combining the optimized features combinations of the ten evaluation repetitions performed for each one of the twenty typically developing subjects. Hence, the highest possible CBOFF value is equal to 200. Moreover, the PBOFIF values obtained for the shoulder flexion game-based exercise, the shoulder horizontal abduction/adduction game-based exercise, and the shoulder adduction game-based exercise are presented in Figure 6d–f, respectively. Both the commonly selected features obtained by considering the CBOFF metric and the important features obtained by considering the PBOFIF metric indicate that the right arm movements of the typically developing subjects can be classified using the angle-based features only. This finding is different from the results reported in Figure 5, which indicate that both the angle- and joint-based features are needed to classify the right arm movements of the cerebral palsy children. The difference between the combinations of optimized features of the typically developing subjects and the combinations of optimized features of the cerebral palsy children can be attributed to the fact that the typically developing subjects do not suffer from shaky and uncontrolled right arm movements. Despite this difference, the results in Figure 6 show that the commonly selected features and the important features obtained by considering the CBOFF metric and the PBOFIF metric, respectively, for the twenty typically developing subjects are dominated by angle-based features related to the $\vec{EW}$ and $\vec{SE}$ vectors. As described in Section 3.4, the angle-based features that are related to the $\vec{EW}$ and $\vec{SE}$ vectors have played also an important role to classify the right arm movements of the six cerebral palsy children.

## 4. Discussion and Conclusions

The contributions of the current study are three folds. First, a participant-specific, game-specific computerized assessment method is developed to assess the correctness of the right arm movements that are performed during game-based rehabilitation exercises. Second, three game-based rehabilitation exercises, which are designed to target the right arm, have been implemented to evaluate the performance of the proposed computerized assessment method. Third, two groups of participants, which include cerebral palsy children and typically developing subjects, are recruited to perform the game-based rehabilitation exercises and the computerized assessment method is used to assess the correctness of the participants’ right arm movements during the game-playing sessions. For the first group of participants that includes six cerebral palsy children, the classification accuracy, specificity, and sensitivity values achieved by the computerized assessment method are within the range of 71% to 95% and the kappa coefficient values are between 0.45 and 0.86, as shown in Table 3. The classification performance of the computerized assessment method varies based on the cerebral palsy category and severity of the cerebral palsy children. For the second group of participants that includes twenty typically developing subjects, the classification accuracy, specificity, and sensitivity values are within the range of 96% to 99% and the kappa coefficient values are between 0.93 and 0.99, as shown in Table 4. The high classification performance results that are obtained for the typically developing subjects can be attributed to the fact that these subjects have high control of their right arm movements. The results reported in the current study suggest the potential of employing the computerized assessment method to assess the correctness of the movements performed by cerebral palsy patients during their engagement in game-based rehabilitation exercises.

Literature reveals that many previous studies that used game-based rehabilitation for cerebral palsy patients evaluated the effectiveness of the game-based exercises using outcome measures that compare the body structure and function, such as range of motion and weakness of muscles, and the level of activity, such as the ability to perform activities of daily living, before and after the game-based rehabilitation [12]. For instance, the study by Hung et al. [19], which employed a set of Kinect-based games to train the upper limbs of cerebral palsy children, evaluated the improvement in the body structure and function and the activity level using the Quality of Upper Extremities Skills Test (QUEST) [41], the Box and Block Test (BBT) [42], the Melbourne Assessment 2 (MA2) [43], and the ABILHAND-kids score [44] outcome measures. In another study, Zoccolillo et al. [10] employed a group of Kinect-based games for the rehabilitation of cerebral palsy patients and used the QUEST and ABILHAND-kids score measures to compare the body structure and function and the activity level before and after the game-based rehabilitation. Acar et al. [45] have also used the QUEST and ABILHAND-Kids score measures as well as the Jebsen Taylor Hand Function Test [46] and the Functional Independence Measure (WeeFIM) [47] to evaluate the enhancement in the body structure and function and the activity level achieved by using a set of Nintendo Wii games as rehabilitation tools for cerebral palsy patients. Compared with these previous studies, the current study is characterized by the use of a computerized assessment method to achieve automatic evaluation of the movements performed by cerebral palsy patients during their engagement in game-based rehabilitation exercises. Such an automatic, computerized assessment approach can be integrated with the outcome measures that evaluate the improvement in the body structure and function and the activity level to analyze the effectiveness of the game-based rehabilitation exercises and improve their utilization as a complementary tool for conventional cerebral palsy rehabilitation therapy.

Despite the promising classification results that are reported in the current study, further studies are required to improve the capabilities of the proposed computerized assessment method. For example, the performance of the computerized assessment method can be improved by employing machine learning technology to classify the movements performed by cerebral palsy patients. For instance, a support vector machine classifier can be used to classify the angle- and joint-based features extracted using the E-MPGD in a manner similar to the procedure proposed by Alazrai et al. [31] to predict the fall of elderly people. The computerized assessment method can also be extended to support the evaluation of the movements performed by the left arm and the legs. In addition, the computerized assessment method can be expanded to provide kinematic-based evaluation metrics to quantify the movements performed by cerebral palsy patients, such as quantifying the smoothness and range of motion of the movements. In fact, such kinematic-based evaluation metrics have been employed by Ding et al. [48] to quantify the smoothness of the movements carried out by stroke patients and Gaillard et al. [49] to quantify the movements performed by unilateral cerebral palsy children based on the “Be an Airplane Pilot” motion analysis protocol.

Although all six cerebral palsy children included in the current feasibility study had the potential to achieve the range of motion targeted by the three game-based exercises as shown in Figure 3, our proposed game-based rehabilitation system can be adapted to support participants that have higher limitations in the range of motion. In particular, the range of motion targeted by the three game-based exercises can be configured to meet the capability of the participant. Moreover, the subject-specific evaluation criteria employed by the physiotherapist to label the game-playing sessions can be adapted to classify the game-playing sessions as correct or incorrect based on the range of motion and physical skills of the participant. This in turn will enable the computerized assessment method, which is trained based on the physiotherapist’s labeling of the game-playing sessions, to adapt to the range of motion that can be achieved by the participant. Furthermore, for participants that cannot perform the three game-based exercises included in the current study, the proposed game-based rehabilitation system can be expanded to include additional game-based exercises that meet the capabilities of these participants and the computerized assessment method can be extended to support the additional exercises. However, despite the potential of expanding the proposed game-based rehabilitation system to support a wider range of cerebral palsy cases, the system might not be applied for cerebral palsy cases that have high limitations in their physical skills, such as the severe cases in which the participants cannot move their limbs independently.

The analysis reported in the current feasibility study did not investigate the relationship between the age of the participants and the performance of the computerized assessment method. Such analysis, which requires a large number of participants with different age groups, is planned in the future. Moreover, the current study did not investigate the effect of the clinical or motor function outcome changes of the participant, which might occur due to conventional rehabilitation therapy as well as game-based rehabilitation exercises, on the classification performance of the computerized assessment method. Such analysis requires the recruitment of a large number of cerebral palsy children that participate in the study for an extended period. In fact, we are planning to perform such analysis in the future. Another future direction is to extend the experimental evaluations of the proposed computerized assessment method to include a wider range of cerebral palsy types and severity levels. Furthermore, the computerized assessment method proposed in this study has been implemented using MATLAB, which affects the capability to achieve real-time assessment of the participant’s movements during the game-playing sessions. Hence, we are planning to implement the computerized assessment method using the C# programming language or the C++ programming language to achieve real-time, computer-based assessment of the participant’s performance.

Despite the fact that our proposed game-based rehabilitation system is configured to use the Kinect for Windows v2 sensor, the system can be easily reconfigured to employ other motion tracking sensors that track the skeleton joints. For example, our game-based system can be easily reconfigured to use the Azure Kinect sensor (Microsoft Corporation, Redmond, WA, USA) that is launched recently as a new generation of the Kinect sensing technology but with improved tracking capabilities. Our system can also be configured to use motion tracking sensors produced by other manufacturers, such as the Orbbec Astra sensor (Orbbec 3D Technology International Inc., Troy, MI, USA).

## Figures and Tables

**Table 1:** The angle- and joint-based features of the E-MPGD.

Type	Features	Description	Mathematical Formulation
Angle-based features	$\theta_{CP}^{\vec{SE}}\left(k\right)$, $\theta_{TP}^{\vec{SE}}\left(k\right)$, $\theta_{SP}^{\vec{SE}}\left(k\right)$	The angles between the vector $\vec{\mathrm{SE}}$ and the CP, TP, and SP at frame *k*.	$\theta_{P}^{\vec{V}}\left(k\right)={sin}^{-1}\left(\frac{|\vec{V}\cdot \vec{n_{P}}|}{\left|\right|\vec{V}\left|\right|}\right)$, where $\theta_{P}^{\vec{V}}$ is the angle between the vector $\vec{V}$ and the plane P at frame *k*, and $\vec{n_{P}}$ is the normal vector to the plane P.
	$\theta_{CP}^{\vec{EW}}\left(k\right)$, $\theta_{TP}^{\vec{EW}}\left(k\right)$, $\theta_{SP}^{\vec{EW}}\left(k\right)$	The angles between the vector $\vec{\mathrm{EW}}$ and the CP, TP, and SP at frame *k*.	
	$\theta_{CP}^{\vec{WH}}\left(k\right)$, $\theta_{TP}^{\vec{WH}}\left(k\right)$, $\theta_{SP}^{\vec{WH}}\left(k\right)$	The angles between the vector $\vec{\mathrm{WH}}$ and the CP, TP, and SP at frame *k*.	
	$\theta_{\vec{SE}}^{\vec{EW}}\left(k\right)$	The angle between the vectors $\vec{\mathrm{SE}}$ and $\vec{\mathrm{EW}}$ at frame *k*.	$\theta_{\vec{U}}^{\vec{V}}\left(k\right)={cos}^{-1}\left(\frac{|\vec{U}\cdot \vec{V}|}{\left|\right|\vec{U}\left||\cdot |\right|\vec{V}\left|\right|}\right)$, where $\theta_{\vec{U}}^{\vec{V}}\left(k\right)$ is the angle between the vectors $\vec{U}$ and $\vec{V}$ at frame *k*.
	$\theta_{\vec{EW}}^{\vec{WH}}\left(k\right)$	The angle between the vectors $\vec{\mathrm{EW}}$ and $\vec{\mathrm{WH}}$ at frame *k*.	
Joint-based features	$p_{x}^{\mathrm{rE}}\left(k\right)$, $p_{y}^{\mathrm{rE}}\left(k\right)$, $p_{z}^{\mathrm{rE}}\left(k\right)$	The *x*, *y*, and *z* components of the rE joint velocity at frame *k*.	The locations of the rE. rW, and rH skeleton joints at frame *k* are provided by the Kinect sensor and transformed to the body-attached coordinate system.
	$p_{x}^{\mathrm{rW}}\left(k\right)$, $p_{y}^{\mathrm{rW}}\left(k\right)$, $p_{z}^{\mathrm{rW}}\left(k\right)$	The *x*, *y*, and *z* components of the rW joint location at frame *k*.	
	$p_{x}^{\mathrm{rH}}\left(k\right)$, $p_{y}^{\mathrm{rH}}\left(k\right)$, $p_{z}^{\mathrm{rH}}\left(k\right)$	The *x*, *y*, and *z* components of the rH joint location at frame *k*.	
	$v_{x}^{\mathrm{rE}}\left(k\right)$, $v_{y}^{\mathrm{rE}}\left(k\right)$, $v_{z}^{\mathrm{rE}}\left(k\right)$	The *x*, *y*, and *z* components of the rE joint velocity at frame *k*.	$v_{d}^{p}\left(k\right)=\frac{p_{d}^{p}\left(k\right)-p_{d}^{p}\left(k-1\right)}{\Delta t}$, where $v_{d}^{p}\left(k\right)$ is the velocity of skeleton joint *p* along direction *d* at frame *k*, $p_{d}^{p}\left(k\right)$ and $p_{d}^{p}\left(k-1\right)$ are the locations of *p* along direction *d* at frames *k* and k−1, respectively, and Δt is the time step between frames *k* and k−1.
	$v_{x}^{\mathrm{rW}}\left(k\right)$, $v_{y}^{\mathrm{rW}}\left(k\right)$, $v_{z}^{\mathrm{rW}}\left(k\right)$	The *x*, *y*, and *z* components of the rW joint velocity at frame *k*.	
	$v_{x}^{\mathrm{rH}}\left(k\right)$, $v_{y}^{\mathrm{rH}}\left(k\right)$, $v_{z}^{\mathrm{rH}}\left(k\right)$	The *x*, *y*, and *z* components of the rH joint velocity at frame *k*.	
	$a_{x}^{\mathrm{rE}}\left(k\right)$, $a_{y}^{\mathrm{rE}}\left(k\right)$, $a_{z}^{\mathrm{rE}}\left(k\right)$	The *x*, *y*, and *z* components of the rE joint acceleration at frame *k*.	$a_{d}^{p}\left(k\right)=\frac{v_{d}^{p}\left(k\right)-v_{d}^{p}\left(k-1\right)}{\Delta t}$, where $a_{d}^{p}\left(k\right)$ is the acceleration of skeleton joint *p* along direction *d* at frame *k*, $v_{d}^{p}\left(k\right)$ and $v_{d}^{p}\left(k-1\right)$ are the velocities of *p* along direction *d* at frames *k* and k−1, respectively, and Δt is the time step between frames *k* and k−1.
	$a_{x}^{\mathrm{rW}}\left(k\right)$, $a_{y}^{\mathrm{rW}}\left(k\right)$, $a_{z}^{\mathrm{rW}}\left(k\right)$	The *x*, *y*, and *z* components of the rW joint acceleration at frame *k*.	
	$a_{x}^{\mathrm{rH}}\left(k\right)$, $a_{y}^{\mathrm{rH}}\left(k\right)$, $a_{z}^{\mathrm{rH}}\left(k\right)$	The *x*, *y*, and *z* components of the rH joint acceleration at frame *k*.	

**Table 2:** The gender, cerebral palsy category, and cerebral palsy severity of the children included in the first group of participants.

Participant	Gender	Cerebral Palsy	Cerebral Palsy
		Category	Severity
Child 1	Male	Spastic diplegic	Mild
Child 2	Male	Spastic diplegic	Mild
Child 3	Female	Spastic diplegic	Moderate
Child 4	Male	Spastic himiplegic	Moderate
Child 5	Female	Ataxia	Moderate
Child 6	Male	Ataxia	Moderate

**Table 3:** The mean ± standard deviation accuracy, specificity, sensitivity, and kappa coefficient values obtained by the computerized assessment method for the six cerebral palsy children.

Participant	Shoulder Flexion				Shoulder Horizontal Abduction/Adduction				Shoulder Adduction			
	Accuracy	Specificity	Sensitivity	Kappa	Accuracy	**Specificity**	Sensitivity	Kappa	Accuracy	Specificity	Sensitivity	Kappa
	(%)	(%)	(%)		(%)	(%)	(%)		(%)	(%)	(%)	
Child 1	93 ± 3	91 ± 4	95 ± 3	0.86 ± 0.06	92 ± 4	91 ± 4	92 ± 5	0.83 ± 0.07	90 ± 4	89 ± 6	90 ± 4	0.80 ± 0.09
Child 2	91 ± 3	90 ± 4	91 ± 5	0.81 ± 0.05	92 ± 3	93 ± 3	91 ± 4	0.84 ± 0.05	90 ± 3	88 ± 4	92 ± 6	0.81 ± 0.07
Child 3	87 ± 4	87 ± 6	88 ± 3	0.74 ± 0.07	86 ± 3	85 ± 5	87 ± 5	0.72 ± 0.06	86 ± 2	87 ± 3	84 ± 4	0.72 ± 0.05
Child 4	85 ± 3	86 ± 5	84 ± 4	0.70 ± 0.06	87 ± 4	88 ± 5	85 ± 4	0.73 ± 0.07	85 ± 4	85 ± 5	86 ± 6	0.71 ± 0.09
Child 5	78 ± 4	80 ± 6	76 ± 5	0.57 ± 0.08	77 ± 4	79 ± 5	75 ± 5	0.54 ± 0.08	72 ± 4	73 ± 5	71 ± 6	0.45 ± 0.07
Child 6	75 ± 3	78 ± 4	73 ± 4	0.51 ± 0.06	76 ± 3	80 ± 4	73 ± 4	0.53 ± 0.06	73 ± 2	73 ± 4	72 ± 3	0.46 ± 0.05
All children	85 ± 7	85 ± 7	84 ± 9	0.70 ± 0.14	85 ± 7	86 ± 7	84 ± 9	0.70 ± 0.14	83 ± 8	83 ± 8	83 ± 10	0.66 ± 0.16

**Table 4:** The mean ± standard deviation accuracy, specificity, sensitivity, and kappa coefficient values obtained by the computerized assessment method for the twenty typically developing subjects.

Participant	Shoulder Flexion				Shoulder Horizontal Abduction/Adduction				Shoulder Adduction			
	Accuracy	Specificity	Sensitivity	Kappa	Accuracy	Specificity	Sensitivity	Kappa	Accuracy	Specificity	Sensitivity	Kappa
	(%)	(%)	(%)		(%)	(%)	(%)		(%)	(%)	(%)	
Subject 1	98 ± 2	98 ± 2	98 ± 2	0.96 ± 0.03	98 ± 2	99 ± 2	98 ± 2	0.97 ± 0.03	97 ± 2	98 ± 2	97 ± 3	0.95 ± 0.04
Subject 2	98 ± 2	98 ± 2	97 ± 3	0.96 ± 0.04	99 ± 1	99 ± 1	99 ± 1	0.99 ± 0.03	98 ± 2	97 ± 2	99 ± 2	0.96 ± 0.03
Subject 3	99 ± 1	99 ± 1	99 ± 2	0.98 ± 0.03	99 ± 1	99 ± 1	99 ± 2	0.98 ± 0.03	99 ± 1	99 ± 1	99 ± 1	0.99 ± 0.02
Subject 4	98 ± 2	98 ± 2	98 ± 2	0.97 ± 0.04	99 ± 1	99 ± 2	99 ± 1	0.98 ± 0.03	98 ± 2	97 ± 3	98 ± 3	0.95 ± 0.04
Subject 5	98 ± 2	97 ± 3	99 ± 2	0.96 ± 0.05	98 ± 1	98 ± 2	98 ± 3	0.95 ± 0.03	97 ± 2	97 ± 2	97 ± 2	0.94 ± 0.03
Subject 6	98 ± 2	99 ± 2	98 ± 3	0.96 ± 0.05	98 ± 2	98 ± 2	98 ± 2	0.96 ± 0.04	97 ± 1	97 ± 2	98 ± 2	0.95 ± 0.03
Subject 7	99 ± 2	98 ± 3	99 ± 2	0.97 ± 0.04	99 ± 2	99 ± 2	99 ± 2	0.98 ± 0.03	98 ± 2	98 ± 2	97 ± 3	0.95 ± 0.05
Subject 8	99 ± 1	99 ± 1	99 ± 1	0.99 ± 0.03	99 ± 2	99 ± 2	98 ± 2	0.97 ± 0.04	97 ± 3	97 ± 3	98 ± 3	0.95 ± 0.06
Subject 9	98 ± 3	97 ± 3	98 ± 2	0.95 ± 0.05	97 ± 2	97 ± 3	97 ± 2	0.94 ± 0.04	97 ± 3	96 ± 3	97 ± 3	0.94 ±0.06
Subject 10	97 ± 3	98 ± 2	97 ± 3	0.95 ± 0.05	97 ± 3	97 ± 2	97 ± 3	0.94 ± 0.05	97 ± 1	96 ± 2	97 ± 2	0.93 ± 0.02
Subject 11	98 ± 2	99 ± 2	97 ± 3	0.96 ± 0.04	98 ± 1	97 ± 2	98 ± 2	0.95 ± 0.03	97 ± 2	97 ± 2	97 ± 3	0.94 ± 0.04
Subject 12	99 ± 2	99 ± 1	98 ± 2	0.98 ± 0.04	99 ± 2	98 ± 3	99 ± 1	0.97 ± 0.04	98 ± 2	97 ± 3	98 ± 2	0.95 ± 0.04
Subject 13	98 ± 3	97 ± 3	98 ± 3	0.95 ± 0.05	98 ± 2	98 ± 2	97 ± 2	0.95 ± 0.04	97 ± 2	97 ± 3	97 ± 3	0.94 ± 0.05
Subject 14	98 ± 1	98 ± 2	99 ± 2	0.96 ± 0.02	98 ± 2	99 ± 2	98 ± 2	0.97 ± 0.03	98 ± 2	98 ± 2	98 ± 3	0.96 ± 0.04
Subject 15	97 ± 2	97 ± 3	97 ± 2	0.95 ± 0.04	98 ± 2	98 ± 2	98 ± 3	0.96 ± 0.05	98± 2	97 ± 3	98 ± 2	0.95 ± 0.05
Subject 16	99 ± 1	99 ± 2	98 ± 2	0.97 ± 0.03	98 ± 1	99 ± 2	98 ± 2	0.96 ± 0.02	98 ± 1	98 ± 2	98 ± 2	0.96 ± 0.03
Subject 17	98 ± 2	99 ± 2	98 ± 3	0.97 ± 0.04	99 ± 1	99 ± 2	99 ± 1	0.98 ± 0.02	98 ± 2	97 ± 2	98 ± 3	0.95 ± 0.04
Subject 18	96 ± 2	97 ±3	96 ± 3	0.93 ± 0.05	98 ± 2	97 ± 3	98 ± 2	0.95 ± 0.05	96 ± 2	97 ± 3	96 ± 3	0.93 ± 0.05
Subject 19	97 ± 3	97 ± 3	96 ± 3	0.94 ± 0.06	97 ± 2	97 ± 3	97± 3	0.94 ± 0.05	96 ± 2	97 ± 2	96 ± 3	0.93 ± 0.03
Subject 20	98 ± 1	98 ± 2	97 ± 2	0.95 ± 0.03	98 ± 3	98 ± 3	97 ± 4	0.95 ± 0.06	97 ± 2	97 ± 2	97 ± 3	0.93 ± 0.04
All subjects	98 ± 2	98 ± 2	98 ± 3	0.96 ± 0.04	98 ± 2	98 ± 2	98 ± 2	0.96 ± 0.04	97 ± 2	97 ± 2	97 ± 3	0.95 ± 0.04
